# Decoding the role of DNA methylation in allergic diseases: from pathogenesis to therapy

**DOI:** 10.1186/s13578-024-01270-0

**Published:** 2024-07-04

**Authors:** Ruiming Han, Dongdong Zhu, Jichao Sha, Boning Zhao, Peng Jin, Cuida Meng

**Affiliations:** 1 Department of Otolaryngology Head and Neck Surgery, China-Japan Union Hospital of Jilin University, Changchun, China; 2 Jilin Provincial Key Laboratory of Precise Diagnosis and Treatment of Upper Airway Allergic Diseases, Changchun, China; 3 Department of Human Genetics, Emory University School of Medicine, 615 Michael ST NE, Atlanta, GA 30322 USA

**Keywords:** DNA methylation, 5-methylcytosine, 5-hydroxymethylcytosine, Allergy, Ten–Eleven translocation proteins

## Abstract

Allergic diseases, characterized by a broad spectrum of clinical manifestations and symptoms, encompass a significant category of IgE-mediated atopic disorders, including asthma, allergic rhinitis, atopic dermatitis, and food allergies. These complex conditions arise from the intricate interplay between genetic and environmental factors and are known to contribute to socioeconomic burdens globally. Recent advancements in the study of allergic diseases have illuminated the crucial role of DNA methylation (DNAm) in their pathogenesis. This review explores the factors influencing DNAm in allergic diseases and delves into their mechanisms, offering valuable perspectives for clinicians. Understanding these epigenetic modifications aims to lay the groundwork for improved early prevention strategies. Moreover, our analysis of DNAm mechanisms in these conditions seeks to enhance diagnostic and therapeutic approaches, paving the way for more effective management of allergic diseases in the future.

## Introduction

**Fig. 1:**
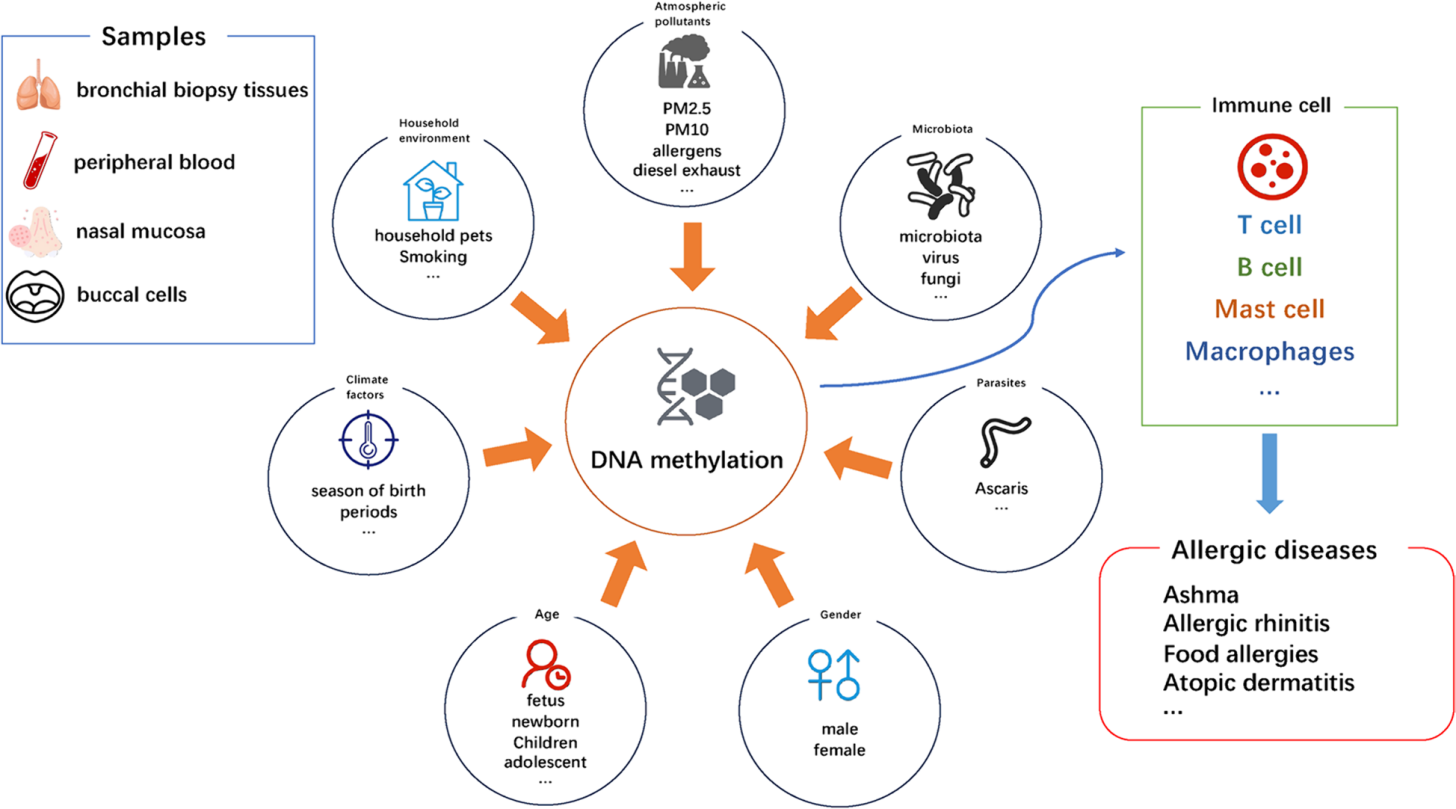
Factors influencing DNA methylation in allergic diseases. The key environmental, biological, and physiological elements that affect DNA methylation are shown, potentially leading to allergic diseases. Key influencers include atmospheric pollutants, household environmental exposures, climate factors, gender, age, parasites, and microbiota. These variables modulate DNA methylation patterns across various immune cells, contributing to the development and exacerbation of allergic conditions. Predominant sources for DNA methylation studies are bronchial biopsy tissues, peripheral blood, nasal mucosa, and buccal cells, highlighting the diverse tissue sampling in current research

Allergic diseases present a diverse array of clinical manifestations and conditions. IgE-mediated allergic diseases are a significant category within this spectrum, commonly known as atopic diseases. These include asthma, allergic rhinitis (AR), and food allergies (FA). Such disorders arise from a complex interplay between genetic and environmental factors. Allergic rhinitis is particularly highly prevalent, significantly impacting patients’ quality of life and socioeconomic development [1]. The prevalence of allergic rhinitis in the population is estimated to be between 10 and 40%, while asthma affects approximately 4.3% [2, 3]. The heritability of asthma is estimated to range from 35 to 70% [4]. Allergic diseases are increasingly understood as resulting from combined genetic and environmental influences [5]. Current research is exploring the connection between epigenetics and allergic diseases [6]. This includes studies on DNAm, histone acetylation, and miRNA level changes in disease pathogenesis. Recent evidence underscores the role of DNAm, an epigenetic modification, in the pathogenesis of allergic diseases. DNAm, involving adding a methyl group to the DNA molecule, is pivotal in maintaining genome stability and regulating gene expression. This review aims to delve deeper into the factors influencing DNA methylation (Fig. 1), examine the relationship between DNAm change and allergic diseases, explore the mechanisms behind DNAm changes, and evaluate their potential impact on preventing and treating these diseases.

## Functions of DNA methylation

Numerous studies have indicated that DNAm plays a critical role in gene expression regulation. It wasn’t until the 1980s that the involvement of DNAm in gene regulation and cell differentiation was demonstrated [7, 8]. Despite the growing volume of DNAm research, understanding the precise functions of many identified DNAm events in the genome remains challenging. DNA methylation involves adding a methyl group to cytosine nucleotides at cytosine-phosphate-guanine (CpG) dinucleotides. These CpG sites often cluster in ‘CpG islands’(CGI) within gene regulatory elements like promoters or enhancers, influencing transcription [9, 10].

The DNA methyltransferase (DNMT) family, comprising key enzymes like DNMT3A, DNMT3B, and DNMT1, play critical roles in DNAm. DNMT3A 、 DNMT3B, and DNMT3L are particularly significant for de novo DNA methylation. DNA methylation is initiated at CpG dinucleotides by the de novo methyltransferases DNMT3A and DNMT3B [11], and is then preserved through cell division by DNMT1 in a replication-dependent manner [12]. In germ cells and embryonic stem cells, the activities of DNMT3A and DNMT3B are further modulated by DNMT3L [13]. The de novo DNA methyltransferase DNMT3A regulates DNA methylation primarily through its PWWP domain, which recognizes histone H3 lysine 36(H3K36) methylation and facilitates the localization of DNMT3A [14, 15]. This is typically absent in CpG islands [16]. And the methylation of CGIs is also regulated by Polycomb repressive complexes [17]. A study suggests that a balance in DNMT3A recruitment by different reader domains, including the PWWP domain recognizing H3K36 methylation, regulates de novo CpG methylation, with ubiquitin-dependent recruitment region (UDR)-mediated recruitment potentially enhanced by oncogenic signaling pathways, leading to CGI hypermethylation patterns observed in various cancer types [18]. DNMT3B employs two flexible loops to encase DNA and its catalytic loop to extrude the cytosine base. It specifically identifies DNA with CpG sites via residues Asn779 and Lys777 in its more stable and orderly target recognition domain loop, facilitating the processive methylation of consecutively repeated CpG sites [19]. DNMT1, on the other hand, predominantly functions to maintain DNAm patterns during cell division. It faithfully replicates methylation patterns from the parental DNA strand to the newly synthesized daughter strand, ensuring epigenetic information is preserved through cell generations.

Converting 5-methylcytosine (5mC) to thymine, spontaneously or through enzymatic deamination, contributes to the loss of CpG sites within the genome. This phenomenon is particularly notable in methylated sequences in the germline, where it can have evolutionary implications [20]. The removal of 5mC, whether passively over cell divisions or actively through enzymatic pathways, is a pivotal regulatory mechanism for gene expression. Since 5mC typically leads to gene suppression, its removal can reactivate previously silenced genes, playing a significant role in gene regulation dynamics [21]. The stable modification of 5mC in mammalian DNA can be oxidized to form 5-hydroxymethylcytosine (5hmC), 5-formylcytosine (5fC), and 5-carboxylcytosine (5caC) by the Ten-Eleven Translocation (TET) protein family, which are Fe^2+^ and 2-oxoglutarate-dependent dioxygenases [22]. TET proteins convert 5mC to 5hmC, 5fC, and 5caC in zygotes, facilitating the active demethylation of paternal DNA [23, 24]. These oxidation products are thought to be diluted passively during preimplantation development, indicating their potential significance at this stage [25]. The stepwise oxidation process, catalyzed by TET proteins, is essential for the dynamic regulation of the epigenetic state of DNA. The TET proteins’ ability to modulate the epigenetic landscape underscores their importance in normal development and the pathogenesis of multiple diseases, including allergic conditions and cancers.

**Fig. 2:**
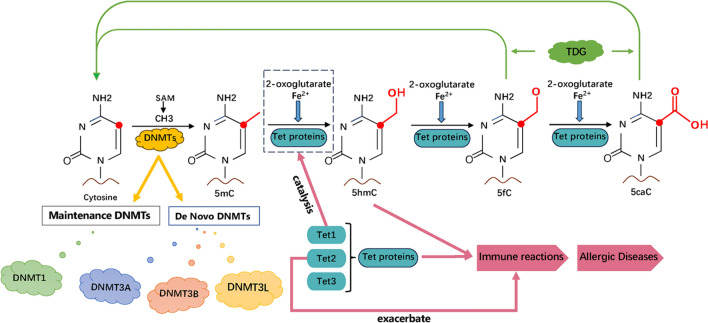
Mechanisms of DNA methylation in allergic diseases. Methylation occurs when cytosine in DNA is converted to 5-methylcytosine (5mC) by DNA methyltransferases (DNMTs), using S-adenosyl methionine (SAM) as a methyl donor. TET proteins can then oxidize the 5mC in the presence of 2-oxoglutarate and Fe^2+^ to produce 5-hydroxymethylcytosine (5hmC), crucial for immune responses. Further oxidation can generate 5-formyl cytosine (5fC) and 5-carboxyl cytosine (5caC), both of which may revert to cytosine through thymine DNA glycosylase (TDG). DNMTs are categorized into maintenance (e.g., DNMT1) and de novo types (DNMT3A, DNMT3B, DNMT3L), while TET proteins (TET1, TET2, TET3) modulate methylation states critical for allergic disease pathogenesis. Specifically, TET1 is noted for its role in converting 5mC to 5hmC in allergic responses, such as allergic rhinitis, with TET2 potentially intensifying these immune reactions

Overall, the intricate dance of methylation and demethylation, facilitated by the DNMTs and TET proteins, respectively, highlights the complexity and dynamism of epigenetic regulation in mammalian cells [22]. Understanding these processes in greater depth could shed light on numerous developmental and pathological phenomena [26, 27] (Fig. 2).

## Factors that affect DNA methylation in allergic diseases

Extensive research into DNAm in allergic diseases has identified various influencing factors. These include environmental elements, microbial and parasitic infections, and demographic variables such as gender and age. Environmental factors are the most comprehensively studied, leading to significant insights into how they affect DNAm in allergic diseases.

### Environmental exposure

#### Atmospheric pollutants

Increasing urban industrialization has elevated atmospheric pollutant levels, correlating with a higher incidence of allergic diseases. DNAm plays a critical role in this relationship. Particulate matter (PM2.5) and diesel fumes are linked to heightened asthma risk and respiratory issues. Studies have established an association between aluminum in PM2.5 and DNAm at asthma gene loci [28]. A comprehensive epigenomic meta-analysis revealed neonatal methylation differences in genes (*USP43*, *SRPRB*, *GNB2L1*; *SNORD96A*, and *P4HA2*) related to airway diseases, suggesting prenatal exposure to PM2.5 and PM10 results in distinct DNAm patterns [29, 30]. Moreover, long-term or repeated exposures to allergens and diesel exhaust (DE) lead to significant changes in DNAm, particularly in the timing and chronicity of exposure [31]. Recent studies have shown that air pollutants can increase DNAm in the promoter region of the FOXP3 gene and the intronic region of the IL10 gene, raising the risk of asthma [32]. The epigenome concept encapsulates gene expression alterations due to environmental factors without DNA sequence changes. Methylation risk scores (MRS) [33] have emerged as biomarkers for sensitization to airborne allergens and future sensitization prediction. In the LISA cohort study, a group of individuals monitored over time, MRS—indices derived from DNAm patterns—were not only correlated with the presence of aeroallergen sensitization, which is the body’s response to airborne allergens, but also exhibited a connection with the actual typical symptoms of allergic diseases [34, 35].

#### Household environmental exposure factors

Epigenome-Wide Association Study (EWAS) on children with asthma/allergic rhinitis showed a correlation between DNAm. Recent studies have uncovered that prenatal smoking by fathers and mothers is differently associated with DNAm patterns in newborns. Joubert et al. observed that connections between maternal smoking and methylation in newborns tend to be more prevalent in island shores and enhancer regions as opposed to promoters or CpG islands [36]. Besides, Kitaba et al. mentioned in their recent study how a father’s smoking, especially if started before they were 15, can affect their children’s DNA. This could increase the kids’ chances of getting asthma or having weaker immune systems. It shows why it’s so important to stop teenagers from smoking—not just for their health but for their future children’s health too [37]. In addition to the aforementioned environmental factors, maternal environmental influences play a significant role in the methylation patterns associated with allergic diseases. Maternal supplementation with L. reuteri during pregnancy modifies DNAm patterns in CD4+ T cells, leading to increased immune activation at birth. This alteration could influence immune maturation and the development of allergies [38, 39].

#### Climate factors

Season of birth influences DNAm, affecting allergic disease risks. For example, DNAm changes associated with birth season correlate with eczema risk by age 18 [40, 41]. Lower Dysregulation of thymic stromal lymphopoietin (TSLP) methylation levels in children relates to respiratory symptoms and increased atopic disease risks [42]. Exposure to butyl benzyl phthalate (BBZP) in settled dust is one of the factors leading to decreased methylation of TSLP. Additionally, the impact of climate factors on DNAm is influenced by spatial effects. A meta-analysis revealed that in any given birth season, there is no overlap in FDR-adjusted differentially methylated CpGs between the high-latitude and low-latitude subgroups [41]. This may be due to regional differences in factors such as sunlight intensity, relative humidity, pollen count, infectious pathogens, and air pollution in different areas.

### Microbiota and parasites

#### Microbiota

Early-life microbiota profoundly influences immune system development and can significantly alter the likelihood of disease development later in life [43, 44]. Studies [45], for instance, have shown that in AR, the microbiota’s impact on epigenetic patterns is enduring. DNAm changes serve as persistent markers of these early-life exposures, potentially influencing gene expression well into later childhood. Such DNAm patterns may interact with the expression of genes pivotal in AR development.

Additionally, rhinovirus infections are known to induce specific DNAm alterations across the genome within the nasal cells of individuals, regardless of their asthma status. These changes notably affect the methylation of immune function-related genes, such as *CXCR4* and *HLA-H*, suggesting that rhinovirus may contribute to the progression of allergic diseases through epigenetic modifications [46].

Furthermore, fungal exposure, particularly to A. fumigatus during late pregnancy as opposed to early exposure, has been associated with distinct DNAm effects in allergic diseases. Such exposure correlates with lower IgE levels and reduced airway eosinophilia in offspring, linked to altered CpG methylation at the IFN-γ and IL-4 gene promoters [47].

#### Parasites

A recent EWAS on Ascaris infection, a condition stemming from soil contamination known to cause respiratory symptoms, involved 671 adult participants from the ‘Respiratory Health in Northern Europe, Spain, and Australia’ study. This research uncovered that seropositivity for Ascaris, indicative of exposure or infection, correlates with distinct methylation patterns in specific genes, including *CRHR1* and *GRK1* [48]. These genes play a role in asthma development, hinting at a potential epigenetic mechanism by which roundworm infections could influence the pathogenesis of asthma.

Additionally, while infection models have observed a correlation between maternal immune responses during schistosomiasis and the increased likelihood of offspring sensitization, they have also demonstrated that acute maternal schistosomiasis does not induce epigenetic modifications in mature gametes. This distinction is crucial for our understanding of infection-related epigenetic phenomena [49, 50].

These insights underscore the significant and potentially long-lasting influence of early microbial environments and parasitic infections on human epigenetics. They emphasize the complex interplay between early life exposures, genetic regulation, and the risk of developing conditions such as asthma. These findings lay the groundwork for future research into preventive strategies and interventions, highlighting the importance of understanding these intricate relationships for better health outcomes.

### Influence of gender, age in early childhood, and gestational factors

Differences in the prevalence of allergic diseases among various groups may be attributed to distinct DNAm patterns, which are known to vary with factors such as gender and age. These epigenetic differences can influence the expression of genes related to immune responses, leading to disparities in susceptibility and severity of allergic diseases across populations. Understanding these methylation patterns is essential for elucidating the epidemiological trends of allergic diseases and customizing their management to suit individual needs.

Naumova and colleagues conducted an analysis of the chromosome region 17q12-21, associated with asthma, and discovered sex-associated variations in DNAm within the *ZPBP2* gene. Beyond the established DNAm differences between males and females in imprinted genes and sex chromosomes, this study reveals more subtle, widespread DNAm patterns linked to gender across the genome [51, 52]. Additionally, significant gender differences have been observed in the methylation of the IFN-γ promoter in patients with allergic asthma. Multivariate regression models indicate that methylation at the CpG -186 site is predictable based on gender [53]. Furthermore, a comprehensive meta-analysis focusing on DNAm in newborns and older children has identified sex-specific differences in additional autosomal loci [54].

The influence of DNAm extends back to the fetal period, suggesting that the aging process impacts the epigenetic landscape from in utero. A comprehensive study encompassing newborns, children, and adolescents has revealed a possible link between maternal hemoglobin levels and DNAm in offspring [55]. Moreover, recent findings demonstrate significant DNAm differences between children aged five who are IgE sensitized to airborne and food allergens and those who are not [56]. These differences have unveiled genes like *RUFY1*, potentially predisposing individuals to such sensitization. Notably, these methylation disparities associated with IgE sensitization are detectable in maternal peripheral blood mononuclear cells (PBMCs), cord blood, and as early as age 2, highlighting early detection and intervention opportunities [56].

Furthermore, the relationship between DNAm and gestational age is evident in preterm infants and full-term infants. This underscores the profound impact of gestational age on infant DNAm levels, reflecting how epigenetic factors vary at different stages of in utero development and potentially influence long-term health outcomes [57, 58].

The interplay between environmental, microbial, parasitic, gender, and age factors and the epigenetic landscape in allergic disease etiology is complex. Understanding these methylation patterns is critical for developing precise interventions and advancing personalized medicine in health.

## DNAm-mediated mechanisms in allergic diseases

### Regulatory role of DNAm in different types of immune cells

Several cell types are fundamentally involved in the evolution and progression of allergic diseases. T cells are vital as they modulate immune responses, produce cytokines, and help maintain the immune system’s equilibrium. B cells also play a significant role, not only by generating antibodies but also through antigen presentation and cytokine secretion. Macrophages influence the trajectory of these diseases with their participation in both inflammatory responses and immune system regulation. Mast cells, too, are essential, as they initiate allergic reactions and are central to the inflammatory process. Collectively, these cell types form the intricate immunological framework characteristic of allergic diseases. In this context, DNA methylation is critically important, affecting the behavior of these immune cells.

#### DNA methylation regulates gene expression in T cells

T cells are crucial for adaptive immunity and become activated upon recognizing peptide-major histocompatibility complex (pMHC) complexes displayed by antigen-presenting cells (APCs). These cells rapidly proliferate and differentiate into various subsets under the influence of the cytokine environment. Among these, CD4+ T helper (TH) cells are central to immune responses, aiding other immune and stromal cells. Upon encountering antigens, CD4+ TH cells, driven by costimulatory signals and cytokines, proliferate and evolve into diverse TH subsets, such as Th1, Th2, Th9, and Th17. Moreover, activated TH cells develop a memory mechanism, enabling swift responses to familiar antigens. Regulatory T cells (Tregs), characterized by their FoxP3 expression, play a role in suppressing immune responses [59, 60]. In the context of allergic diseases, the differentiation of T helper cells is known to be governed by DNAm. Alterations in DNAm patterns within CD4+ T cells have been observed in allergy patients, correlating with changes in CD4+ T cell counts, particularly in conditions like allergic rhinitis [61].

DNAm significantly impacts CD4+ T cells, affecting activation and repressing *IFNG* gene expression during experimental asthma. It adds to the adaptability of CD4+ T cell populations [62]. Research by Standing et al. in a mouse asthma model showed that *GLI* activity induction in leukocytes lowers CD4+ Th2 cell recruitment to allergic lungs [63]. *GLI2*’s enhanced expression affects T-cell receptor (TCR) repertoire selection, altering the CD4+ /CD8+ ratio [64]. Another hypothesis posits that increased *GLI2* gene methylation might reduce TGF-β1 levels, leading to greater differentiation of Th immune cells and increased production of pro-inflammatory cytokines [65].

The transcription factor FOXP3 is essential for CD4+ regulatory T (Treg) cell development and function [66]. FOXP3 expression during Treg cell differentiation is regulated by conserved noncoding sequence (CNS) elements [67]. Tan et al.’s study on allergic rhinitis in mice suggested maternal allergic disorders can result in offspring with abnormal immune states linked to Foxp3 promoter region hypermethylation [68].

Furthermore, DNAm is critical in defining TH cell subtypes. Without DNMT3A, an enzyme crucial for de novo methylation, there is misexpression of the Th1 cytokine IFN-γ in other CD4+ T cell subtypes [69]. The methylation levels of *AIM2* and *CCL5* genes in memory CD4 cells decrease significantly [70]. Nestor et al. showed that *AIM2* and *CCL5* undergo 5-hydroxymethylcytosine remodeling during immune memory activation development [71].

#### DNA methylation controls how genes are expressed in B cells

DNAm in B cells is also critical in allergic diseases. Alterations in the DNAm patterns of B cells can lead to irregular gene expression, impacting immune responses and contributing to allergic reactions [72–74]. Notably, children with food allergies often exhibit a higher proportion of circulating B cells than non-allergic (NA) children, a trend particularly evident in both naïve and memory B-cell populations (including switched and non-switched types) [75]. Additionally, variations in the populations of regulatory B cells (Bregs) in allergic individuals underline the significance of B cells in modulating allergic responses. This role is exemplified by the ability of Breg-derived interleukin IL-10 to inhibit the release of Th2 cytokines [76].

Key to the differentiation of memory B cells are the gene loci *PRDM1* and *BACH2*. Zhang et al.’s study identified distinct DNAm levels at these loci, differentiating between naïve and memory B cells [70]. The *AIM2* locus, crucial for developing B cell immune memory, also exhibits significant methylation differences [77]. Furthermore, notable changes in DNAm have been observed at the *BCL2* locus, further underscoring the dynamic epigenetic landscape of B cells in allergic conditions [78].

#### The role of DNA methylation in mast cells

Mast cells are known for their direct role in releasing inflammatory factors and their interactions with T cells and B cells. These interactions are crucial in balancing immune responses and inflammatory reactions. Recent studies have shown that the DNMT inhibitor, 5-aza-2′-deoxycytidine, can modulate the effects of these interactions. Interestingly, these effects are reversible with the removal of IL-6. This suggests that methylation changes at the *SOCS3* promoter influence the regulation of *SOCS3*, leading to increased phosphorylation and activation of *STAT3* by IL-6 [79]. Additionally, it has been observed that elevated expression of DNMT1 results in further methylation of the *SOCS3* promoter. However, the specific mechanisms behind these processes in the context of allergic diseases remain an area for further investigation.

#### The impact of DNA methylation on macrophages

Macrophage polarization plays a vital role in the development of asthma, influenced by local micro-environmental factors. Macrophages recruited to the site can differentiate into classically activated (M1) or activated (M2) phenotypes [80]. There is a significant correlation between DNAm and the differential expression of genes in M1 and M2 macrophages. The enzymes DNMT1, DNMT3A, and DNMT3B are particularly noteworthy, as they exhibit distinct expression patterns in these macrophage types and are key in gene silencing [81]. Interestingly, a reduction in DNMT3B levels favors the M2 macrophage phenotype associated with decreased inflammation [82]. Conversely, increasing DNMT3B levels promote a shift towards the M1 phenotype. The mechanism behind this involves DNMT3B‘s interaction with the methylation region of PPARγ1, a critical transcription factor that regulates macrophage polarization [83].

### Mechanisms of immune response mediated by ten-eleven translocation (TET) proteins and 5-hydroxymethylcytosine

TET enzymes and 5hmC are believed to be critical in immune responses to antigens, particularly in allergic diseases [26, 27]. The roles of 5fC and 5caC in these diseases are unclear, but there is growing evidence linking TET enzymes and 5hmC to antigen-induced immune reactions [84]. Studies have found elevated levels of TET enzymes and global 5hmC in peripheral blood mononuclear cells (PBMCs) of patients with AR, along with increased TET1 expression in dendritic cells [26, 85]. Changes in 5hmC levels could be due to shifts in TET1 binding across the genome or a complex regulatory mechanism involving transcriptional upregulation of TET1 and inhibition of its enzymatic activity by oxidative stress from external exposures [86, 87].

Our previous studies indicate that TET2 deficiency exacerbates ovalbumin-induced AR in mice, impacting major histocompatibility complex (MHC) molecules, regulators, and ligands [88]. This deficiency also alters 5hmC levels at immune response genes, affecting adaptive immunity and cytokine expression. Observations by Tan et al. on global changes in 5hmC and TET2 expression in CD4 + T cells from AR patients revealed lower global DNA 5hmC deposition and TET 2 expression compared to healthy individuals [89]. This deficiency in TET2 was linked to more severe allergic inflammation. Furthermore, a decrease in TET2 expression was associated with increased methylation at the FOXP 3 Treg-specific demethylated region (TSDR), reduced FOXP 3 expression, and fewer Treg cells in AR, suggesting that TET 2 may regulate Treg cell function by modulating FOXP3 methylation [90].

These studies highlight the crucial role of TET enzymes, particularly TET1 and TET2, and the DNA modifications they induce, such as 5hmC, in allergic disease development and immune response. They underscore the complex interplay between epigenetic changes and immune regulation, offering new insights for understanding and treating allergic disorders.

### Exploring the role of DNA methylation in allergic diseases through epigenome-wide association studies

In allergic diseases, changes in the methylation patterns of specific CpG sites can affect the expression of genes that regulate immune responses, inflammation, and biological pathways related to allergic reactions. Understanding these methylation changes is crucial for unraveling the pathogenesis of allergic diseases and could aid in diagnosis and treatment. However, most studies examining the human methylome are limited, as epigenotyping arrays used for comprehensive DNAm analysis cover only about 450,000 to 840,000 of the approximately 29 million CpG sites in the human genome, representing just 1.6% to 2.9% of the total [91]. These epigenetic markers may directly influence disease risk or interact with environmental factors, offering insights that complement genome-wide association studies (GWAS).

In epigenomic studies, methylated cytosine on CpG dinucleotides is a common focus. With the advent of array-based DNAm measurement platforms, there’s been a surge in EWAS. For example, a study on U.S. Project Viva children identified various CpG sites and differentially methylated regions (DMRs) linked to asthma and allergies, particularly impacting eosinophil and TH2 cell activities [92]. Decreased methylation in genes regulating these activities was noted, with specific changes associated with neutrophil degranulation and IL-4 and IL-13 pathways. Another study highlighted that CpG methylation levels between 20% and 80% are most strongly associated with asthma and allergic predisposition, with methylation in gene-coding regions particularly relevant [93].

In an Epigenome-Wide Association Study involving 2,286 asthma patients, numerous CpG sites displayed differential methylation in non-atopic asthma, implicating 382 new genes [94]. A larger number of CpG sites were found in atopic asthma, involving 569 novel genes, with an overlap of 104 CpG sites between atopic and non-atopic asthma [94]. These CpG sites indicate T cell and macrophage migration, critical contributors to allergic inflammation. A specific study identified a DMR within the regulatory sequence upstream of the *PM20D1* gene, linking increased methylation (hypermethylation) in this region to wheezing in early childhood [95]. Another comprehensive review linked nine CpG sites and 35 DMRs in umbilical cord blood to the risk of developing asthma in school-aged children [96]. High methylation of the *GLI2* gene in the saliva of these children is associated with IL4 and TGF-β1 dependent inflammation pathways. A DMR in the *GLI2* gene, situated in a CpG shore encompassing several enhancers and promoters, was identified as a distinguishing factor between children with respiratory allergies and healthy controls [97].

In the context of food allergies, Safar et al. summarized previous research and identified 464 differentially methylated genes linked to food allergies [98]. CpG oligodeoxynucleotides (CpG ODNs), synthetic DNA sequences with unmethylated CpG motifs, activate the innate immune system through Toll-like receptor 9 (TLR9) signaling, stimulating immune responses like the production of pro-inflammatory cytokines and enhancing antibody production [99]. A study showed that CpG-ODN has a protective effect against ovalbumin-induced allergic airway inflammation, potentially related to the inhibition of the JNK signaling pathway, as demonstrated in mouse models and in vitro studies using RAW264.7 cells [100].

## Applications of DNAm in allergic diseases

### DNAm in the diagnosis of allergic diseases

Increasing evidence highlights the pivotal role of DNAm in diagnosing and understanding the progression of allergic diseases. Family history is a notable factor in developing these diseases, and recent studies have underscored its importance in predicting asthma’s risk and severity [101]. Nasal DNAm has emerged as a valuable biomarker, distinguishing between symptomatic and asymptomatic IgE sensitization [102] (Table 1). The application of DNAm in precision medicine, particularly in conditions like eosinophilic asthma, is promising. Combining methylation profiles with genomic data allows personalized treatments to be developed, leading to more effective therapies [103]. Epigenetic aging concepts could transform disease progression tracking, enabling earlier interventions. van Breugel et al. proposed a model based on methylation at three nasal CpG sites that can predict allergic diseases in adolescents, with validation in a separate cohort. This finding demonstrates the potential of DNAm as a biomarker and indicates its capability to detect multiple allergic conditions [102].

**Table 1:** Application of DNAm in allergic diseases

	Method	Application	Applicable people	Allergic disease	Study
Diagnosis	Collecting nasal brushing samples from the lower part of the inferior turbinate; Detecting the following three CpG sites in the sample: cg20372759, cg01870976, and cg24224501	Predicting allergic diseases	Childhood	Asthma/allergic rhinitis/eczema	Merlijn van Breugel, et al. [102]
	Differential analysis of DNAm in airway smooth muscle cells obtained from bronchial biopsies	Assessing disease severity	Not specified	Asthma	Mark M. Perry,et al. [104]
	By analyzing DNAm in buccal cells and nasal epithelial cells	Diagnosing diseases	Children	Asthma	Rossa Brugha,et al. [107]
	Identifying differentially methylated sites associated with asthma severity in nasal epithelial cells	Predicting disease severity	Children	Asthma	Tao Zhu,et al.2021 [108]
Therapeutic	Determining the CpG methylation sites in the promoter and intron regions of the T cell Foxp3 gene in blood	Assessing the efficacy of immunotherapy	People who receive sublingual immunotherapy	Allergic rhinitis	Ravi S. Swamy, et al. [110]
	Administering CpG-ODN injections	Alleviating airway inflammation	Not specified	Asthma	Hai-Yun Zhang,et al. [100]
	Genomic DNA was extracted from peripheral blood mononuclear cells of patients who underwent SIT to determine DNAm levels and gene expression	Personalized therapy	Children who received SIT	Asthma	Chuang-Ming Wang,et al. [113]

Lower airway biopsies are effective for studying asthma-related biological processes. Unique DNAm patterns, correlated with asthma occurrence and severity, have been identified. These patterns are linked to gene and miRNA expression changes, influencing airway smooth muscle cells [104]. However, such methods are invasive and challenging, particularly in children or during flare-ups. Additionally, DNAm specificity to tissues and cell types adds complexity [105]. Recent studies suggest nasal epithelium as a surrogate for lower airway epithelium in asthma, offering a less invasive biomarker [106, 107]. Zhu et al. found significant DNAm differences in children with varying asthma severities, with specific CpG sites linked to clinical features, highlighting their potential in assessing asthma severity [108].

In AR, allergen exposure leads to rapid epigenetic changes in peripheral blood cells, characterized by increased DNAm. This responsiveness to allergens suggests that peripheral blood-based methods could effectively diagnose AR [109].

### Therapeutic applications of DNAm research

Reduced allergen sensitivity, following immunotherapy, is associated with decreased DNAm at the FOXP3 gene locus, enhancing regulatory T cell roles [110, 111]. This suggests DNAm’s role in building allergen resistance. Sublingual immunotherapy in AR patients also leads to decreased methylation at the FOXP3 locus, indicating DNAm’s significance in AR development. Monitoring DNAm changes could aid in assessing immunotherapy effectiveness.

In Puerto Rican children with asthma, albuterol modified DNA methylation in significant CpG sites and genomic regions, influencing genes related to asthma pathways [112]. This suggests a genetic predisposition to methylation changes, offering a basis for personalized albuterol treatment assessment. CpG ODNs show potential as vaccine adjuvants, cancer immunotherapies, and treatments for allergic diseases by modulating immune responses. They might serve as adjunctive therapy for allergic airway inflammation. Allergen-specific immunotherapy (SIT) with Dermato-phagoides pteronyssinus protease (Der p) alters the DNA methylomes of PBMCs, potentially affecting genes involved in inflammation reduction. Understanding these changes could enhance allergy treatment tailoring [113] (Table 1).

While the potential therapeutic applications of DNAm in allergic diseases are promising, they remain speculative, necessitating further research for validation [114, 115]. Exploring DNAm mechanisms is crucial for personalized treatment approaches in these conditions.

## Summary and future perspectives

In this review, we present a comprehensive overview of the current research landscape regarding the factors and mechanisms of DNAm in allergic diseases. This discussion underscores the significant role of environmental factors in influencing the DNAm patterns associated with these diseases.

While substantial progress has been made in understanding the mechanisms by which DNAm influences allergic diseases, translating this knowledge into practical applications is still at its infancy. The field stands on the cusp of breakthroughs that could revolutionize the diagnosis and management of allergic conditions. DNAm research holds tremendous potential in developing diagnostic tools that are more precise and less invasive. These advancements could lead to earlier detection of allergic diseases, enabling more timely and effective interventions. Furthermore, the potential therapeutic applications of DNAm research in allergic diseases are vast and diverse. As we unravel the complex interplay between genetics, epigenetics, and environmental factors, we can anticipate novel therapeutic approaches that target specific epigenetic modifications. These therapies could offer personalized treatment strategies, potentially improving patient treatment response with various allergic conditions.

Looking ahead, it is evident that DNAm research will continue to be a vital field of study. Future research should focus on expanding our understanding of the epigenetic mechanisms in allergic diseases. This will involve integrating DNAm studies with other omics approaches, such as genomics, transcriptomics, and proteomics, to provide a more holistic view of disease pathogenesis. Additionally, longitudinal studies tracking DNAm changes over time in response to environmental exposures and therapeutic interventions will be crucial in elucidating the dynamic nature of epigenetic regulation in allergic diseases.

In conclusion, DNAm research in allergic diseases is a rapidly evolving field with the potential to impact how these conditions are diagnosed and treated significantly. As we continue to explore and understand the complexities of epigenetic regulation, the future holds promise for more personalized and effective management of allergic diseases.

## Data Availability

Not applicable.
